# The therapeutic potential of attentional bias modification training for insomnia: study protocol for a randomised controlled trial

**DOI:** 10.1186/s13063-018-2937-4

**Published:** 2018-10-19

**Authors:** Umair Akram, Bronwyn Milkins, Antonia Ypsilanti, John Reidy, Lambros Lazuras, Jodie Stevenson, Lies Notebaert, Nicola L. Barclay

**Affiliations:** 1 Department of Psychology, Sociology and Politics, Sheffield Hallam University, Collegiate Crescent, Sheffield, South Yorkshire S10 2BP UK; 2 University of Western Australia, Elizabeth Rutherford Memorial Centre for the Advancement of Research on Emotion, Perth, Australia; 3 Department of Psychology, The University of Sheffield, Sheffield, UK; 4 Sleep and Circadian Neuroscience Institute, Nuffield Department of Clinical Neurosciences, Sir William Dunn School of Pathology, University of Oxford, Oxford, UK

**Keywords:** Insomnia, Attentional bias, Attention bias modification, ABM, Cognitive bias, Randomised controlled trial

## Abstract

**Background:**

The efficacy of attentional bias modification (ABM) as a treatment for anxiety and depression has been extensively studied with promising results. Despite some evidence of sleep-related attentional biases in insomnia, only a small number of studies, yielding mixed results, have examined the application of ABM in insomnia. This study specifically aims to determine whether ABM can reduce (i) the presence of an attentional bias for sleep-related threatening words; (ii) insomnia symptom severity; (iii) sleep onset latency; and (iv) pre-sleep cognitive arousal amongst individuals with insomnia compared to a non-treatment control group of individuals with insomnia.

**Methods/design:**

We propose a randomised controlled trial of 90 individuals from the general population who meet the criteria for Insomnia Disorder. Following an initial examination for the presence of a sleep-related attentional bias using the dot-probe paradigm, participants will be randomised to an online attentional bias modification training condition, or to a standard attentional bias task (non-treatment) control condition. Both conditions will be delivered online by a web platform. All participants allocated to the non-treatment control group will be offered ABM training once the study is complete. The primary outcome will be the attentional bias indices of vigilance and disengagement and self-reported insomnia symptoms, sleep onset latency and pre-sleep cognitive arousal. Attentional bias and insomnia symptoms will be assessed at baseline (day 1) and post-treatment (2 days after the final training session: day 9). Insomnia symptoms will be again assessed at follow-up (day 16). Secondary outcomes include examining whether sleep associated monitoring and worry are related to a sleep-related attentional bias in insomnia, and whether such reports reduce following ABM. All main analyses will be carried out on completion of follow-up assessments. The trial is supported by the Department of Psychology, Sociology and Politics at Sheffield Hallam University.

**Discussion:**

This study will extend the research base examining the efficacy of attentional bias modification for insomnia.

**Trial registration:**

ISRCTN (ISRCTN11643569, registered on 5 June 2018).

## Background

Cognitive models of insomnia propose that selective attention for sleep-related information acts as a maintaining factor of the disorder [1, 2]. Such attention may be placed on internal (i.e. bodily sensations) or external (i.e. outside noises) cues and may occur on awakening, during the day and in the pre-sleep period. It is theorized that selective attention for sleep-related information contributes to increased physiological and cognitive arousal, and excessive worry concerning the negative daytime consequences (i.e. impaired daytime functioning) which are perceived to precede night-time sleep difficulty. These processes are said to generate and maintain sleep-specific anxiety, which ultimately produces a state that is incompatible with the initiation of sleep. In support of this notion, several studies have investigated attentional biases to sleep-related cues in insomnia using experimental reaction time (i.e. dot-probe, flicker, and emotional Stroop) and eye-tracking paradigms [3–12]. The majority of these studies suggest that poor sleepers and individuals with insomnia display attentional biases towards sleep-related words and images compared with normal-sleepers [3–5, 7–13].

In disorders such as anxiety and depression, where attentional biases are prominent, it has been evidenced that attention biases for negative, disorder salient, information can be alleviated using attentional bias modification (ABM) tasks [14]. In these studies, attentional avoidance of negative information is encouraged through use of a modified dot-probe task where probes always appear in the location opposite negative stimuli. Over time, this procedure has been known to ‘train’ an individual’s attention away from negative information related to their specific condition [14]. When applied prior to an event perceived as threatening to a specific population (i.e. anxious individuals asked to speak in public), the immediate effects of ABM appear to be most prominent [15].

Recent research has examined the efficacy of delivering ABM immediately prior to bed as a treatment for individuals reporting elevated disposition to experiencing poor sleep quality [16, 17]. Specifically, these studies obtained evidence that individuals provided with ABM also reported improved subjective sleep quality, reduced pre-sleep arousal, and reduced sleep onset latency relative to control conditions where a standard attentional bias task was delivered. However, these studies utilised a measure of disposition to experiencing poor sleep to recruit participants, rather than a measure of disposition to experiencing insomnia-specific symptoms, which limits the generalisability of these studies. To that end, Lancee et al. [18] explored the therapeutic effect of ABM amongst those meeting diagnostic criteria for insomnia. Whilst no therapeutic effects were observed in this study, ABM was administered in the evening between 7 and 11 pm, rather than immediately prior to bed when biased attention may be most prominent [16].

The present research aims to further examine the therapeutic potential of attentional bias modification for the treatment of insomnia-specific symptoms. Previous research has proved to be efficacious where delivery of ABM and control tasks are alternated amongst the same sample [16]. Whilst this research highlights promising outcomes for those experiencing sleep disturbance, these outcomes cannot be extrapolated to individuals with insomnia. Moreover, the within-subjects alternating nature of this research may also prevent any potential accumulation of improved sleep from being otherwise observed. Therefore, the current study will implement a between-subjects design using a sample of individuals meeting the diagnostic criteria, who will be randomly allocated to either a treatment or non-treatment control condition. This will allow for observation of ABM over repeated nights and for the presence of any potential cumulative changes in outcome measures. Specifically, using a similar modified dot-probe task to Milkins et al. [16], we aim to determine whether ABM can reduce (i) the presence of an attentional bias for sleep-related negative words characterised by either vigilance (i.e. speeding of attention towards salient stimuli) or disengagement (i.e. difficulty in shifting attention away from salient stimuli); (ii) insomnia symptom severity; (iii) sleep onset latency; and (iv) pre-sleep cognitive arousal amongst individuals with insomnia compared to a non-treatment control group of individuals with insomnia. As a secondary aim, we also seek to determine whether initial attentional bias is related to greater self-reports of sleep-associated monitoring behaviour and sleep-related worry, and whether such reports reduce following ABM.

## Methods/design

### Trial design

**Fig. 1:**
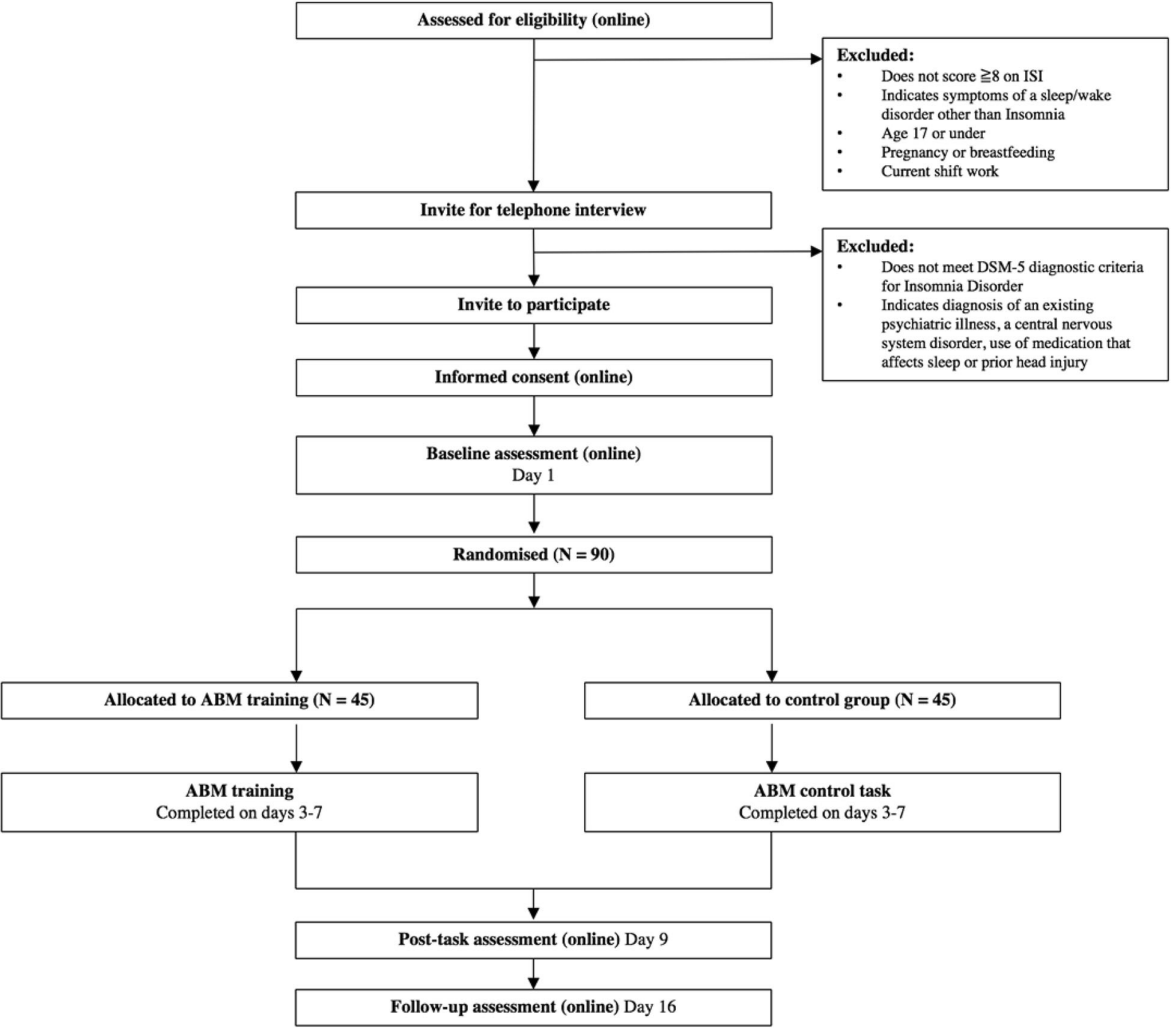
Flow chart diagram showing the summary of the trial design. *DSM-5* Diagnostic and Statistical Manual of Mental Disorders, 5^th^ edition, *ISI* Insomnia Severity Index

This study is a double-blind randomised controlled trial examining the efficacy of ABM for insomnia delivered over the internet (see Fig. 1. for summary). The study has received ethical approval from the Sheffield Hallam University Research Ethics Committee (reference number: ER5451619).

### Participants and recruitment

We will recruit a total of 90 adult members from the general population who meet the *Diagnostic and Statistical Manual of Mental Disorders, 5*^*th*^ *edition* (DSM-5) criteria for Insomnia Disorder [19]. Potential participants will be recruited through the website ‘call for participants’ and various insomnia treatment forums and will be assessed online for eligibility. Those interested in taking part will complete the Insomnia Severity Index (ISI: [20]), and SLEEP-50 [21] measures (see ‘Questionnaire Measures’ section for more detail). Those indicating subclinical insomnia using the ISI, and do not indicate the presence of another sleep/wake disorder other than insomnia on the SLEEP-50 will be invited for a telephone interview to determine the presence of DSM-5 Insomnia Disorder. Telephone interviews will be conducted by the principal investigator (UA).

Specifically, individuals with insomnia must report dissatisfaction with sleep characterized by either a difficulty initiating or maintaining sleep or early morning awakenings. Insomnia should be present for 3 or more nights per week, for at least 3 months, and cause significant daytime impairment. Finally, these conditions must be met despite an adequate opportunity to sleep. Those who report symptoms of a sleep/wake disorder other than insomnia, an existing psychiatric illness, a central nervous system disorder, use of medication that may affect sleep, a prior head injury, pregnancy or breast-feeding or current shift-work will be ineligible to take part. Those meeting the criteria for Insomnia Disorder will be invited to participate and will be provided with an email link for the online study. All participants must have normal to corrected vision.

### Randomisation and allocation concealment

This study will use a simple randomisation with an allocation ratio of 1:1 which will be carried out by the automated online system. As such, the research team will not be able to influence randomisation and will not have access to future allocations.

### Blinding

This will be a double-blind trial. Once invited to participate, all assessments and group allocation will occur online, and the research team will be blind to outcomes during the trial. Participants will not be aware whether they are in the experimental or control condition. It is relevant to note that the research team is unlikely to have any contact with the participants, and as such it will be unlikely that they are able to bias allocation or assessment.

### Word pairs

#### Assessment pairs

In this study we will use the same set of 48 sleep-negative and neutral word pairs as used by Milkins et al., ([16]; see Tables 1 and 2 for word pairs). A subset of 24 of these sleep-negative and neutral word pairs will be used alongside 24 neutral-neutral word pairs. Each of the words will be matched on frequency and length.

**Table 1:** List of sleep-negative/neutral and neutral-neutral word pairs for attentional bias assessment points (baseline and post-treatment)

Sleep-negative and neutral pairs		Neutral-neutral pairs	
Sleep-negative word	Matched neutral word	Neutral word	Matched neutral word
Ache	Melt	Graft	Males
Lethargy	Teaspoon	Featuring	Scrubbing
Agitated	Carriers	Cushion	Specify
Naps	Kits	Meals	Chord
Angry	Curve	Led	Lot
Overactive	Clearances	Stairs	Mirror
Coffee	Beside	Invoice	Prodigy
Sleepy	Wallet	Playful	Tramped
Delirious	Fragments	Margin	Pencil
Snoring	Penguin	Tops	Laps
Disastrous	Curriculum	Book	News
Tense	Trick	Habit	Steel
Disorder	Compost	Sandals	Potters
Sluggish	Decorate	Attendants	Speculates
Distress	Creature	Invents	Drafted
Restless	Receiver	Graft	Disarm
Exhausted	Developed	Point	Water
Snoring	Penguin	Thankful	Entrants
Hopeless	Borrowed	Vaccine	Embrace
Sleepy	Wallet	Wriggles	Fixation
Inadequate	Initiative	Pear	Bath
Nightmare	Volunteer	Trait	Stand
Irritable	Appliance	Days	Motel
Drowsy	Umpire	Eraser	Places

**Table 2:** List of sleep-negative/neutral word pairs for ABM experimental and control conditions

Sleep-negative and neutral pairs		Neutral-neutral pairs	
Sleep-negative word	Matched neutral word	Sleep-negative word	Matched neutral word
Ache	Melt	Lazy	Hint
Afraid	Artist	Lethargy	Teaspoon
Agitated	Carriers	Medication	Bottleneck
Alarm	Spray	Naps	Kits
Angry	Curve	Nightmare	Volunteer
Anxiety	Express	Overactive	Clearances
Coffee	Beside	Panicky	Infuser
Conflict	Advanced	Restless	Receiver
Delirious	Fragments	Sad	Cup
Desperate	Aesthetic	Sick	Pull
Disastrous	Curriculum	Sleepless	Advisable
Disengaged	Nonfiction	Sleepy	Wallet
Disorder	Compost	Sluggish	Decorate
Distract	Brochure	Snoring	Penguin
Distress	Creature	Stress	Links
Drowsy	Umpire	Suffer	Branch
Exhausted	Developed	Tense	Trick
Fatigue	Passive	Time	Look
Hopeless	Borrowed	Tired	Plain
Illness	Mustard	Unpleasant	Expedition
Inadequate	Initiative	Wakeful	Trolley
Irrational	Flashlight	Work	Life
Irritable	Appliance	Workload	Handbags
Jittery	Thermos	Worry	Theme

*ABM* attention bias modification

#### ABM control and experimental pairs

Here, we will use the full set of 48 sleep-negative and neutral word pairs as used by Milkins et al., [16].

### Questionnaire measures

#### Primary measures

##### Insomnia severity

Insomnia symptoms will be assessed using the Insomnia Severity Index [20] (ISI). The ISI consists of seven items examining the severity of insomnia symptoms over the past 2 weeks including difficulty initiating and maintaining sleep and awakening too early. Items are scored on a 5-point Likert scale, with total scores ranging from 0 to 28. Higher scores represent greater insomnia severity, with scores of ≥ 8 indicative of subclinical insomnia.

##### Consensus sleep diary

The critical measure of sleep onset latency (SOL) will be assessed as part of a standardized sleep diary [22], completed by participants each morning of the study. Here, participants will record in minutes the estimated time taken to fall asleep the previous night.

##### Pre-sleep cognitive arousal

To assess pre-sleep cognitive arousal before and after delivery of the ABM/control tasks, we will use the Pre-Sleep Arousal Scale (PSAS: [23]). The scale contains 16 items related to the kinds of thoughts and sensations experienced in the pre-sleep period which can contribute to sleeplessness.

#### Secondary measures

##### Sleep associated monitoring

Monitoring will be assessed using the Sleep Associated Monitoring Index (SAMI: [24]), a 30-item measure comprising of eight subscales: daytime monitoring for body sensations; calculation of time; waking monitoring for body sensations; pre-sleep monitoring for body sensations consistent with falling asleep; daytime monitoring of functioning; pre-sleep monitoring for body sensations inconsistent with falling asleep; pre-sleep monitoring the clock; and pre-sleep monitoring the environment. Items are measured on a 5-point scale on which participants indicate what is true for them over the past week (1 = not at all, 5 = all the time). Mean scores for subscales reflect total subscale scores divided by the number of items in the scale.

##### Anxiety and preoccupation with sleep

Anxiety and sleep-related worry will be examined using the Anxiety and Preoccupation about Sleep Questionnaire (ASPQ: [25]) and the Sleep Anticipatory Anxiety Scale (SAAQ: [26]). The ASPQ is comprised of 10 items that examine the extent to which participants have been worried about the nature and consequences of their sleep problem over the past 3 days. The SAAQ consists of 10 items that examine sleep-related worry and physiological aspects of anticipatory anxiety.

#### Assessment points and intervention

##### Baseline attentional bias assessment and questionnaire measures

At baseline (day 1), after blind group allocation, all participants will complete an initial measure of sleep-related attentional bias using a dot-probe task consisting of the neutral and sleep-negative word pairs. The attentional bias assessment will be delivered online using Inquisit 3.0.3.2 software (Millisecond Software, Seattle, WA, USA).

**Fig. 2:**
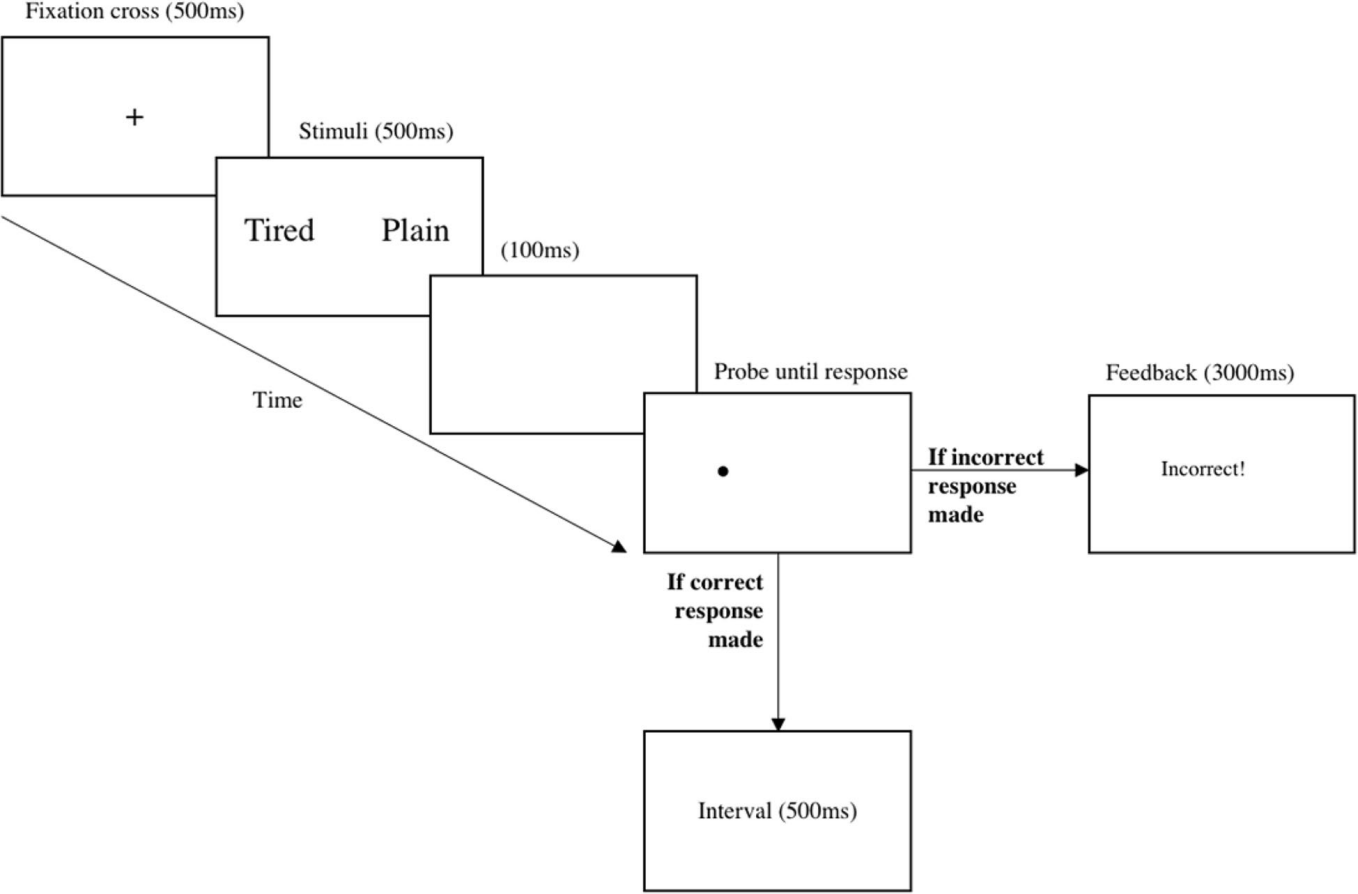
Example order of individual trial sequence for the dot-probe task. Fixation cross first presented for 500 msec, followed by word presentation side-by-side for 500 ms. After 100 ms interval, probe appears until response. If the corresponding probe location key (i.e. correct response) was pressed, no feedback is presented, and the next trial begins after 500 ms blank screen interval. However, incorrect response feedback is presented if the wrong key is pressed triggering a 3000 ms interval before the next trial begins

The dot-probe assessment task will consist of 384 trials in total, with 10 repetitions of 48 word pairs delivered in randomised blocks (24 sleep-negative/neutral, and 24 neutral/neutral pairs at five repetitions each). In both the experimental and control condition, each trial starts with a fixation cross appearing in the centre of the screen for 500 ms, followed by presentation of a word pair vertically aligned (3 cm separation), appearing for 500 ms. After a gap of 100 ms, a dot-probe (two small dots aligned either horizontally or vertically) appears either in the top or bottom position, remaining on the screen until a response key was pressed. Participants are specifically required to press the corresponding key, which indicated the position of the probe (key Z for left position; M for right position), as quickly and as accurately as possible. If participants responded incorrectly, error feedback will be provided triggering a 3 s delay. If a correct response is made, the screen will be cleared, and the next trial will commence after an interval of 500 ms (see Fig. 2). Here, for the initial and follow-up attentional bias assessments, the probes replace the negative and neutral member of each word pair with equal frequency to allow for the examination of vigilance and disengagement for sleep-negative stimuli.

The modified ABM version of the task will consist of 10 repetitions of 48 word pairs delivered in randomised blocks (all sleep-negative/neutral). Here, the probe always replaces the locus of the neutral word, promoting attentional avoidance of sleep-negative stimuli. In the non-ABM control condition, all task parameters are identical to the modified ABM version, however the probes replaced the negative and neutral member of each word pair with equal frequency without promoting attention toward or away from negative words.

Following this, participants will complete: the ISI, SAMI, ASPQ, and SAAQ.

##### ABM intervention and control group procedure

On days 3–7, using a computer, participants will be required to complete a dot-probe task prior to bed. The nature of the dot-probe task will depend on group allocation. Those in the experimental treatment condition will be provided with a modified version of the aforementioned dot-probe task such that the probe only appears in the location of the neutral word. Theoretically shifting (training) their attention away from the sleep-negative words. Whereas those in the control condition will complete the same task with probes replacing the negative and neutral member of each word pair with equal frequency. Following the task, participants will complete the Pre-Sleep Arousal Scale prior to bed. On awakening, participants will be required to complete an online version of the consensus sleep diary.

##### Post ABM assessment and questionnaire measures

Following this 5-per week protocol, all participants will the complete the same attentional bias task (as completed at baseline) on the final day of the experimental phase (day 9). Moreover, the same questionnaire measures (ISI, SAMI, ASPQ, SAAQ) and sleep diary will also be completed.

##### Follow-up assessment

At follow-up (day 16) participants will again complete the ISI, SAMI, ASPQ and SAAQ. On completion, participants will be provided with an Amazon e-gift voucher for taking part. Moreover, those in the control condition will be invited to take part in the experimental treatment if willing.

#### Sample size rationale

The current power estimation was based on the meta-analysis on ABM training for anxiety conducted by Hakamata and colleagues [27] who noted a mean effect size equal to Cohen’s *d* = .61. In each group, we would require 44 participants based on a *t* test, power of .80 and an alpha level of .05 (two-tailed).

#### Statistical analyses

##### Examination of accuracy and preparation of attentional bias data

Only data from correct trials will be included in the final analysis. In addition, reaction times below 200 ms, those above 3000 ms, and those that are three standard deviations above each participant’s individual mean will be eliminated [28].

##### Calculation of vigilance and disengagement

To examine attentional bias, indices of vigilance and disengagement will be calculated. The vigilance index is calculated by subtracting the mean reaction time for negative stimuli from the mean reaction time for neutral stimuli: Vigilance index = dN,N – dT, N. Here, dN, N represents dots replacing neutral words in the presence of another neutral word (i.e. neutral: neutral-neutral trials); and dT, N for dots replacing sleep-negative words in the presence of a neutral word (i.e. congruent: sleep-neutral trial). A positive score on the vigilance index indicates faster response to dots appearing after sleep-related as compared to neutral words [24], i.e. greater vigilance for ‘negative’ stimuli. To calculate disengagement from sleep-related words, the mean reaction time for neutral trials are subtracted from the mean reaction time for trials where the dots replaced neutral stimuli in the presence of sleep-related stimuli: Disengagement index = dN, T – dN, N. Here, dN, T represents dots replacing neutral words in the presence of a sleep-related word (i.e. incongruent: sleep-neutral trials). A positive score on the disengagement index indicates slower responses to dots replacing neutral words in the presence sleep-related words [24], i.e. that attention was not directed to the neutral word but directed elsewhere (possibly the sleep-related word) when the sleep-related word is present. In sum, greater positive scores for both indices, vigilance and disengagement, are indicative of an attentional bias.

##### Analyses

First, we will examine group differences in attentional bias indices at baseline with the expectation that there should be no group difference. Following this, we will examine differences in attentional bias indices at follow-up (i.e. day 9). To examine whether any attentional bias reduced amongst the experimental group, we will then examine the group (experimental versus control) × time (baseline versus post-ABM) interaction for each index of attentional bias (i.e. vigilance and disengagement). Second, to examine whether insomnia symptoms reduced amongst the experimental group, we will examine the group (experimental versus control) × time (baseline versus post-ABM versus follow-up) interaction. Third, to separately examine whether pre-sleep cognitive arousal and sleep-onset latency reduced amongst the experimental group on nights where the tasks were completed, we will examine the group (experimental versus control) × time (days 3 vs. 4 vs. 5 vs. 6 vs. 7) interactions. Finally, to explore the secondary aim we will employ a series of Pearson’s correlational analyses to determine whether each of the attentional bias indices (i.e. vigilance and disengagement) are related to each subscale of the SAMI. Simple effects analyses will be performed to decompose any significant interactions as appropriate.

#### Dissemination

We will publish the results of this study in a peer-reviewed journal, regardless of the magnitude or direction of the results. Findings may also be presented at both national and international scientific meetings. The data will be made available online where possible, if permitted by journal policies.

**Fig. 3:**
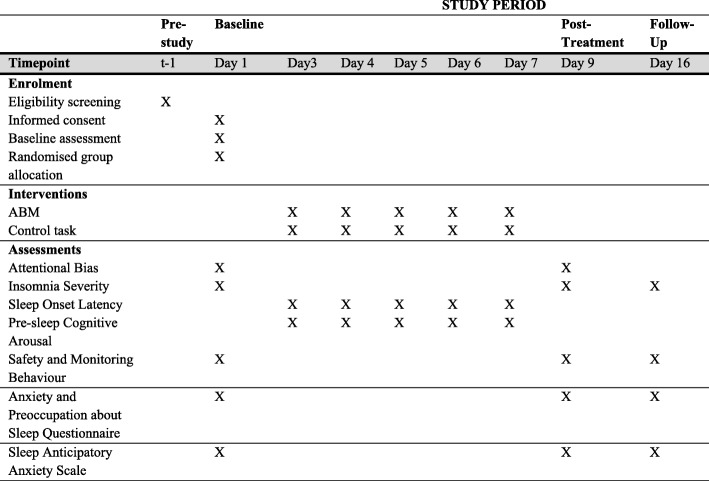
Standard Protocol Items: Recommendations for Interventional Trials (SPIRIT) figure

Items in this protocol comply with the Standard Protocol Items: Recommendations for Interventional Trials (SPIRIT) Checklist (see Fig. 3 for the SPIRIT figure).

#### Potential limitations

It is relevant to note the procedural limitations that might arise during data collection. Considering the 16-day length of this protocol, we anticipate a degree of participant dropout. In addition, considering the online delivery of ABM, some participants may experience technical difficulties in the absence of an experimenter.

## Discussion

Sleep-related attentional biases are considered to be a key factor in the development and maintenance of insomnia [1, 2]. This study will determine whether attentional bias modification can be used to reduce the presence of an attentional bias, and whether this reduces symptom severity amongst individuals with insomnia.

### Trial status

Recruitment of participants commenced on 10 May 2018 and is ongoing. Data collection will begin 1 November 2018.

## Abbreviations

ABM: Attentional bias modification
APSQ: Anxiety and Preoccupation about Sleep Questionnaire
DSM-5: *Diagnostic and statistical manual of mental disorders, 5* ^*th*^ *edition*
ISI: Insomnia Severity Index
PSAS: Pre-Sleep Arousal Scale
SAAS: Sleep Anticipatory Anxiety Scale
SAMI: Sleep Associated Monitoring Index
SOL: Sleep onset latency
